# Dual mechanisms of a purified root extract of *Paeonia lactiflora* Pall. in regulating uric acid homeostasis: inhibition of hepatic synthesis and promotion of renal excretion in hyperuricemic rat models

**DOI:** 10.3389/fphar.2026.1800063

**Published:** 2026-04-07

**Authors:** Tingting Jin, Ruoling Xu, Zhuoxin Jiang, Juncheng Ma, Ning Li

**Affiliations:** Anhui Key Laboratory of Bioactivity of Natural Products, School of Pharmacy, Anhui Medical University, Hefei, China

**Keywords:** *Paeonia lactiflora* Pall, hyperuricemia, xanthine oxidase, adenosine deaminase, renal transporter protein, uric acid

## Abstract

**Introduction:**

Hyperuricemia (HUA), characterized by persistently elevated serum uric acid (UA), represents a growing global public-health concern. Current urate-lowering medications are often associated with hepatotoxicity and nephrotoxicity, highlighting the urgent need for safer, food-derived alternatives.

**Methods:**

A macroporous resin-purified fraction (PLE-2) from *Paeonia lactiflora* Pall. root extract was characterized by HPLC fingerprinting and evaluated in HK-2 human renal proximal tubular epithelial cells and a potassium oxonate/hypoxanthine-induced rat model of HUA.

**Results:**

*In vitro*, PLE-2 exhibited significant cytoprotective activity against UA-induced injury. *In vivo*, oral administration of PLE-2 (100, 200, and 400 mg/kg) dose-dependently reduced serum UA levels to ranges comparable with allopurinol, while attenuating renal injury (decreased creatinine and blood urea nitrogen, improved kidney histology). The mechanism involves dual regulation: suppression of hepatic UA production via inhibition of xanthine oxidase (XOD) and adenosine deaminase (ADA), and enhancement of renal UA excretion through downregulation of reabsorption transporters (URAT1, GLUT9) and upregulation of secretion transporters (OAT1, OAT3, ABCG2), confirmed by qPCR and Western blot. Molecular docking showed strong binding of paeoniflorin (the major metabolite in PLE-2) to these targets.

**Discussion:**

PLE-2 demonstrates potent dual action on UA synthesis and excretion with notable nephroprotective effects, positioning the purified fraction as a promising functional food metabolite for preventing and ameliorating HUA.

## 1 Introduction

Hyperuricemia (HUA) is a chronic metabolic disorder characterized by persistently elevated serum UA levels, resulting from disturbances in purine metabolism (Li et al., 2020; Liu et al., 2024). It is widely recognized as the physiological basis of gout and is closely associated with the pathogenesis of chronic kidney disease, cardiovascular diseases, and metabolic syndrome (Dalbeth et al., 2016; Maiuolo et al., 2016). UA homeostasis is maintained by the balance between hepatic production and renal excretion. Xanthine oxidase (XOD) and adenosine deaminase (ADA) are the key enzymes responsible for UA synthesis in the liver (Fang et al., 2024; Li et al., 2025). XOD acts as the rate-limiting enzyme, catalyzing the oxidation of hypoxanthine and xanthine to UA, while ADA contributes by converting adenosine and deoxyadenosine into inosine and deoxyinosine, respectively. Inhibiting XOD and ADA represents a viable therapeutic strategy for HUA.

Renal excretion accounts for approximately 70% of total UA elimination and is mediated by a complex network of transporters in the proximal tubule, including reabsorption and secretion transporters (Bobulescu and Moe, 2012; Mandal and Mount, 2015). The kidney serves as the primary regulatory organ for UA handling, involving four sequential processes: glomerular filtration, proximal tubular reabsorption, secretion, and post-secretory reabsorption (Cai et al., 2020). Key reabsorptive transporters include urate transporter 1 (URAT1) and glucose transporter 9 (GLUT9). URAT1, localized to the apical membrane of proximal tubular cells, facilitates UA reabsorption from the tubular lumen into the cells, playing a major role in maintaining serum UA levels (Enomoto et al., 2002; Wu et al., 2015). UA secretion is primarily mediated by organic anion transporters 1 and 3 (OAT1 and OAT3) on the basolateral membrane, which transport UA from the blood into tubular cells (Granados and Nigam, 2024; Nigam et al., 2015), and ATP-binding cassette subfamily G member 2 (ABCG2) on the apical membrane, which actively secretes UA into the tubular lumen (Woodward et al., 2009; Yamagishi et al., 2010). Thus, modulating the expression and function of these transporters, in addition to enzyme inhibition, offers a promising approach to regulate UA excretion and systemic homeostasis.

Currently, the clinical management of HUA relies heavily on synthetic agents such as allopurinol (an XOD inhibitor) and benzbromarone (a uricosuric agent) (FitzGerald et al., 2020; Zhang et al., 2018). Although effective, these drugs are frequently associated with severe adverse effects, including hepatotoxicity, nephrotoxicity, and hypersensitivity reactions (Wu et al., 2021; Xie et al., 2014). Consequently, there is a growing trend toward exploring safe and effective anti-HUA agents from natural functional foods or “medicine-food homology” materials, which are believed to exert multi-target effects with minimal side effects (Lu et al., 2022).

*Paeonia lactiflora* Pall., a member of the Ranunculaceae family, has been recorded since the Shennong Ben Cao Jing as a classic “medicine-food homology” botanical drug in China, serving both therapeutic and dietary purposes (Gu et al., 2024). As a classic embodiment of the concept of “medicine and food homology” in China, it serves both therapeutic and dietary purposes. As a dietary metabolite, it offers gentle, sustained support for conditions such as blood deficiency and liver qi stagnation (Guo N. et al., 2025; Hall et al., 1990). Crucially, its traditional application in “clearing heat and cooling blood” (Qingre Liangxue) provides a theoretical foundation for mitigating systemic inflammation and protecting organ function Modern pharmacological studies have confirmed that the total glycoside fraction of this botanical drug exhibits anti-inflammatory and UA-lowering effects in HUA models, demonstrating advantages over synthetic drugs through its multi-target action and lower toxicity (Guo J. W. et al., 2025; Kang et al., 2020).

However, conventional water extraction methods often lead to loss of active metabolites or introduction of excessive impurities, limiting efficacy and consistency. To address these limitations, the present study employed macroporous resin purification to obtain a standardized fraction (PLE-2) enriched in key monoterpene glycosides (particularly paeoniflorin), which was then characterized by HPLC fingerprinting. The hypouricemic and nephroprotective potential of PLE-2 was evaluated using integrated *in vitro* (HK-2) and *in vivo* (potassium oxonate/hypoxanthine-induced rat model) approaches, combined with molecular docking, to elucidate its dual-regulatory mechanism on UA synthesis and renal handling.

## 2 Materials and methods

### 2.1 Chemicals and materials

AB-8 macroporous adsorption resin was provided by Macklin Biochemical. The model establishment involved the acquisition of potassium oxnate (P831461) and hypoxanthine (H811076) from Macklin Biochemical. Nanjing Jiancheng Biotechnology Institute supplied assay kits used for the determination of UA, creatinine (CRE), blood urea nitrogen (BUN), XOD and ADA. Protein antibodies URAT1 (14937-1-AP), GLUT9 (67530-1-Ig), ABCG2 (27286-1-AP) and OAT1 (26574-1-AP) were obtained from Wuhan Sanying Biotechnology Co., Ltd. Protein antibodies OAT3 (PA5-102699) were acquired from Thermo Fisher Scientific-CN. Anti-XOD (ab109235) and Anti-ADA (ab108352) were acquired from Abcam. AG RNAex Pro Reagent, Evo M-MLV RT Premix, SYBR Green Premix Pro Taq HS Kit (Guangzhou Ruizhen Biotechnology Co., Ltd.), and Sangon-synthesized primers, and their sequences are listed in Supplementary Table S1 of the Supplementary Material.

### 2.2 Preparation of *P*. *lactiflora* extracts

*P*. *lactiflora* was provided by Anhui Xiehecheng Chinese botanical drug Co., Ltd. (Bozhou, Anhui Province, China) in September 2023. This medicinal material is native to Bozhou City, Anhui Province, China. Its identification was performed by Prof. Kai-Jin Wang from Anhui University (batch number: 23101201). A voucher specimen has been deposited in the Natural Medicinal Chemistry Laboratory, School of Pharmacy, Anhui Medical University, for future reference.

The roots of 1,000 g of *P. lactiflora* were subjected to systematic extraction by overnight maceration in 6 L of 95% ethanol. The mixture was subjected to reflux extraction at 60 °C for 2.5 h, followed by vacuum filtration using a Buchner funnel. The filtrate was concentrated by rotary evaporation (58 °C, 45 rpm). This procedure was repeated sequentially with 85% and 70% ethanol. The resulting filtrates were pooled and further concentrated into a thick paste, designated as the ethanolic fraction (PLE).

The remaining residue was subjected to water decoction twice. The pooled aqueous extract was concentrated and then subjected to water extraction followed by alcohol precipitation to isolate crude polysaccharides, which were lyophilized into a powder (PLP). PLE fraction was reconstituted in an appropriate volume of water and homogenized by ultrasonication. The suspension was loaded onto a macroporous resin column and eluted with an ethanol-water gradient. Fractions were monitored using thin-layer chromatography and combined according to their chemical profiles. Four distinct subfractions were obtained, lyophilized, and designated as PLE-1, PLE-2, PLE-3, and PLE-4.

### 2.3 HPLC fingerprint analysis and peak identification of PLE-2

To establish the chemical profile of the bioactive fraction, 100 mg of PLE-2 was dissolved in 1 mL of methanol, homogenized, and filtered through a 0.22 μm membrane prior to HPLC analysis.

Separation was performed on an Atlantis analytical column with the following standardized parameters: detection wavelength of 210 nm, 230 nm, 254 nm, 280 nm, with a flow rate of 1.0 mL/min and an injection volume of 10 μL. The gradient elution program was as follows: 0–40 min, B/A from 5:95 to 15:85; 40–90 min, B/A from 15:85 to 25:75; 90–110 min, B/A from 25:75 to 100:0.

### 2.4 In vitro

#### 2.4.1 Cell culture

Human renal tubular epithelial HK-2 cells were obtained from the American Type Culture Collection (ATCC). Cells were maintained in DMEM/F-12 medium supplemented with 10% heat-inactivated fetal bovine serum and 1% penicillin–streptomycin solution, under standard culture conditions (37 °C, 5% CO_2_, and >95% humidity).

For revival, cryopreserved HK-2 cells were rapidly thawed in a 37 °C water bath with gentle agitation until only a small ice crystal remained. The cell suspension was immediately transferred to a centrifuge tube containing pre-warmed complete medium, centrifuged at 1,200 r/min for 4 min, and the supernatant carefully aspirated. Pelleted cells were washed twice with sterile phosphate-buffered saline (PBS), resuspended in fresh complete medium, and seeded into flasks. After 24 h, the medium was replaced to remove non-adherent cells and residual dimethyl sulfoxide (DMSO). When the cell confluence reaches 70%–80%, subculture is performed: the culture medium is aspirated, the cells are rinsed twice with sterile PBS, and then incubated with 0.25% trypsin-EDTA solution at 37 °C for 60–90 s. When the cells become round and detach in large areas under a phase contrast microscope, an equal volume of complete medium with serum is added to terminate the digestion. The resulting suspension is gently pipetted to obtain a uniform single-cell suspension and subcultured at a ratio of 1:2 or 1:3. For functional assays, exponentially growing HK-2 cells were seeded into 96-well plates at a density empirically determined to yield ∼70% confluence after 24 h. To minimize edge effects, 100 μL sterile PBS was added to all outer wells. Plates were incubated for 24 h prior to experimental treatments to ensure uniform cell attachment and monolayer formation.

#### 2.4.2 Cell viability assays

After cell attachment, the culture medium was removed, and HK-2 cells were seeded at a density of 5 × 10^3^ cells/well in 96-well plates and cultured overnight to allow for complete attachment. Cells were then randomly assigned to the normal control (NC), model control (MC), or PLE-2 treatment groups. The model group was only treated with UA and no drug therapy was administered. The treatment groups were pre-treated with PLE, PLP, PLE-1, PLE-2, PLE-3, and PLE-4 at final concentrations of 1.25, 2.5, 5, 10, and 20 μg/mL. After 24 h of pre-treatment, the supernatant was discarded. HUA was induced in the MC and treatment groups by exposure to UA (100 μmol/L), while the NC group was maintained in fresh medium.

After drug treatment for the predetermined duration, the culture medium was aspirated from each well of the 96-well plate. Cells were gently washed once with sterile PBS to remove residual medium and drugs. Subsequently, 100 μL of serum-free DMEM/F-12 medium containing 10% CCK-8 reagent was added to each well to ensure uniform coverage of the cell monolayer without bubble formation. Plates were incubated in a humidified 37 °C, 5% CO_2_ incubator for 2 h until a clear colorimetric change was observed. Following incubation, absorbance was measured at 450 nm using a microplate reader, with blank wells used as background controls. Three technical replicates were included per concentration group, and the experiment was independently repeated three times.

#### 2.4.3 Western blot analysis

Experimental groups include the normal control (NC), model control (MC), AP (50 μmol/L) as a positive control, and PLE-2 treatment groups at low, medium, and high concentrations (5, 10, and 20 μg/mL, denoted as D1, D2, and D3, respectively). Cellular proteins were isolated from each group of HK-2 cells at low temperature using RIPA lysis buffer. The supernatant was collected for subsequent analysis by Western blotting.

Protein samples were separated by SDS-PAGE and subsequently transferred onto polyvinylidene fluoride membranes. After a 2 h blocking step in 5% skim milk, the specific primary antibodies were diluted in Tris-buffered saline containing 0.1% Tween-20 (TBST) and incubated overnight with the following membranes: ADA (1:5,000), XOD (1:5,000), URAT1 (1:1,000), GLUT9 (1:10,000), OAT1 (1:1,000), OAT3 (1:1,000), ABCG2 (1:5,000), and GAPDH (1:5,000). The membranes were then washed three times with TBST, followed by incubation with horseradish peroxidase (HRP)-conjugated rabbit or mouse secondary antibodies (1:5,000) at room temperature for 1 h. Finally, the membranes were washed three additional times with TBST, and protein bands were visualized using an enhanced chemiluminescence detection kit and analyzed with ImageJ software (Zhang et al., 2023).

### 2.5 In vivo

#### 2.5.1 Animal grouping and treatment

This study received approval from the Laboratory Animal Ethics Committee of Anhui Medical University (Protocol No. LLSC20242193; Approval Date: September 2024).

Thirty-six male SPF Sprague-Dawley rats (200 ± 20 g) were randomly assigned to six groups (n = 6): the normal control (NC), model control (MC), and positive treatment group (AP, 10 mg/kg), and D1, D2, and D3 represent 100, 200, and 400 mg/kg of PLE-2. HUA was induced by intraperitoneal injection of potassium oxonate (300 mg/kg) and hypoxanthine (100 mg/kg) dissolved in 0.5% CMC-Na. The treatment groups received daily oral gavage starting 1 h after induction for 14 consecutive days.

#### 2.5.2 Biochemical assays

Two hours after the final dose, rats were anesthetized and blood was collected from the abdominal aorta. Serum was separated by centrifugation at 5,000 rpm for 15 min at 4 °C. The levels of serum UA, CRE, BUN, and XOD were determined according to the manufacturer’s instructions provided with the assay kits. Liver and kidney tissues were rapidly excised, rinsed with 0.9% saline, and either stored at −80 °C for further analysis or fixed in 4% paraformaldehyde for histological examination. Tissue homogenates (10% w/v) were prepared in ice-cold saline to measure XOD and ADA activities.

#### 2.5.3 Western blot analysis

The tissue protein was extracted from approximately 50 mg of rat kidney samples, which were carefully weighed and homogenized at low temperature in RIPA buffer containing PMSF and phosphatase inhibitors. The following steps are described in Section 2.4.3.

#### 2.5.4 RNA extraction and quantitative Real-Time PCR

Total RNA was extracted from rat kidney tissues using TRIZOL reagent as described above. The concentration and purity of the RNA were assessed, and reverse transcription was performed to synthesize cDNA. Subsequently, qPCR was carried out on a Bio-Rad CFX96 Real-Time PCR Detection System. The reaction mixture (10 μL) contained SYBR Green Master Mix, cDNA template, gene-specific primers, and nuclease-free water. The cycling protocol included an initial denaturation at 95 °C for 30 s, followed by 40 cycles of denaturation at 95 °C for 5 s and annealing at 60 °C for 30 s. Relative mRNA expression levels were determined using the comparative Ct (2^−ΔΔCT^) method and normalized to GAPDH.

#### 2.5.5 Histopathological examination

Fixed kidney samples were dehydrated, embedded in paraffin, and sectioned into 4-μm slices. Sections were stained with Hematoxylin and Eosin (H&E) according to standard protocols and imaged using a digital slide scanner to evaluate pathological changes (Wang et al., 2021).

### 2.6 Molecular docking

Molecular docking was then performed to predict binding modes between paeoniflorin and seven HUA-relevant targets: XOD, ADA, URAT1, GLUT9, OAT1, OAT3, and ABCG2. Crystal structures of human target proteins were sourced from PDBe-KB and processed in PyMOL 2.5.5 by removing water molecules, co-crystallized ions, and native ligands. The paeoniflorin structure was energy-minimized using the MMFF94 force field, and both ligand and receptor files were converted to PDBQT format using ADFR Suite 1.0. Docking simulations employed AutoDock Vina 1.2.3 with a broad search space (40 × 40 × 40 Å^3^) covering the entire protein surface, exhaustiveness = 32, and default settings for all other parameters. Binding poses were ranked by predicted binding affinity (ΔG, kcal/mol), and the top-scoring pose for each target was visually validated and analyzed in PyMOL 2.5.5.

### 2.7 Statistical analysis

Analysis data are presented as mean ± standard deviation (SD). Statistical significance was determined by one-way analysis of variance (ANOVA) followed by Tukey’s *post hoc* test using SPSS software. A value of P < 0.05 was considered statistically significant.

## 3 Results

### 3.1 Chemical profiling and standardization of PLE-2

**FIGURE 1:**
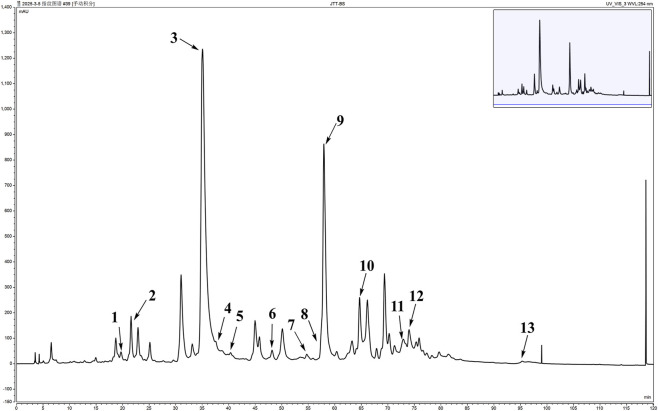
HPLC fingerprint analysis of PLE-2 and reference standards. This chromatogram displays the chemical profile of PLE-2 with major characteristic peaks identified by comparing retention times with standard substances. The peaks are numbered as follows: 1, Methyl gallate; 2, Catechin; 3, Paeoniflorin; 4, Ethylgallate; 5, 8-Hydroxyphellandral; 6, 6′-(2-hydroxypropanoyl)-paeoniflorin; 7, Acetoxypaeoniflorin; 8, Ethyl 3,4-dihydroxy-5-methoxybenzoate; 9, 1.2.3.4.6-*O*-pentagalloylglucose; 10, Paeonidanin; 11, Terephthalic acid bis(2-ethyl-hexyl) ester; 12, 4-*O*-methylpaeoniflorin; 13, Benzoylpaeoniflorin.

Four fractions, namely, PLE-1 (0.45 g), PLE-2 (57.61 g), PLE-3 (0.52 g), and PLE-4 (6.51 g), were obtained using macroporous resin chromatography. The fraction from the PLE-2 extraction site exhibited the highest abundance, with an extraction yield of 5.76%. After comprehensive evaluation of both target metabolite sensitivity and overall chromatographic information content, 254 nm was selected as the final detection wavelength. Results obtained at other wavelengths are provided in Supplementary Material (Supplementary Figures S18–S20) As shown in Figure 1, the baseline of the chromatogram of PLE-2 was stable, and the peaks of each metabolite were well separated, indicating that the macroporous resin purification process was effective. Among the identified metabolites, paeoniflorin (peak 3) had a relative peak area of 32% and a retention time of about 35 min, with the largest relative peak area, which was consistent with its role as the main bioactive marker in *P*. *lactiflora*. The presence of these substances indicated that the enrichment method effectively retained the key active metabolites in the crude medicinal materials (Ji et al., 2013; Zhang and Wei, 2020).

### 3.2 PLE-2 protects HK-2 cells against high UA-induced injury

**FIGURE 2:**
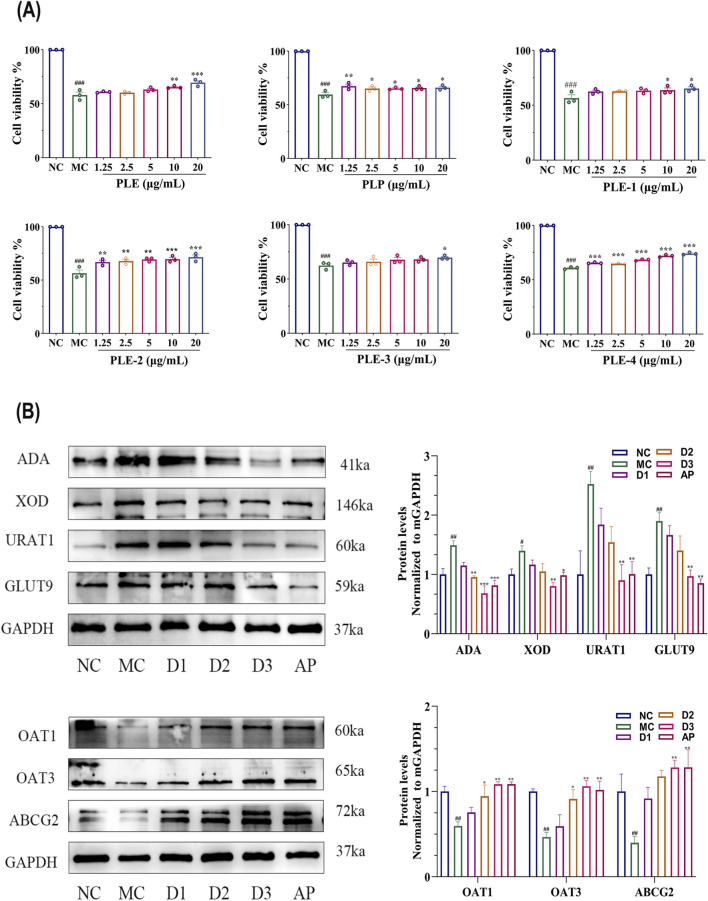
Combined assessment of cell viability and target protein expression indicates the superior bioactivity of PLE-2. **(A)** Impact of PLE, PLP, PLE-1, PLE-2, PLE-3, and PLE-4 on the viability of HK-2 cells. **(B)** Effects of PLE-2 and AP on the expression levels of enzymes and transport proteins involved in UA synthesis, including ADA, XOD, URAT1, GLUT9, OAT1, OAT3, and ABCG2 in HK-2. The NC groups, MC groups, AP groups (50 μmol/L), and treatment groups at concentrations of 5, 10, and 20 μg/mL (D1, D2, D3) were evaluated. Data are from three independent experiments and expressed as mean ± SD (n = 3). Statistical analysis was performed using one-way ANOVA followed by Tukey’s *post hoc* test. Compared with the NC group, #p < 0.05, ##p < 0.01, ###p < 0.001; compared with the MC group, *p < 0.05, **p < 0.01, ***p < 0.001.

Figure 2A shows the cytoprotective effects of six distinct extract fractions of *P lactiflora*. on HK-2 under hyperuricemic conditions. As shown, compared with the normal control group, cell viability in the model group was significantly reduced (P < 0.001), confirming the pronounced cytotoxic effects of elevated UA levels on HK-2. Following pharmacological intervention, varying degrees of recovery in cell viability were observed in all treatment groups. All fractions exhibited a clear dose-dependent protective effect: as the concentration increased from 1.25 μg/mL to 20 μg/mL, cell survival rates showed a progressive increase. Notably, the PLE, PLE-2, and PLE-4 fractions showed the most potent and consistent cytoprotective effects; at 20 μg/mL, cell viability in these groups was restored to levels approaching those of the normal control group. Importantly, PLE-2 exhibited significant cytoprotective activity even at the low concentration of 1.25 μg/mL (P < 0.01), indicating high biological potency.

Figure 2B shows that in terms of UA synthesis-related enzymes, compared with the blank control group, the protein expression levels of ADA and XOD in MC group were significantly increased (P < 0.01). The three concentration groups of PLE-2 dose-dependently downregulated the protein expression of ADA and XOD, with the high concentration group (20 μg/mL) showing the most significant inhibitory effect (P < 0.001). For UA transport proteins, URAT1 and GLUT9 expression was significantly increased in the model group (P < 0.01) and decreased after PLE-2 treatment (P < 0.01). PLE-2 also upregulated OAT1 (P < 0.01), OAT3 (P < 0.001), and ABCG2 (P < 0.001) in a dose-dependent manner.

### 3.3 Biochemical detection

**FIGURE 3:**
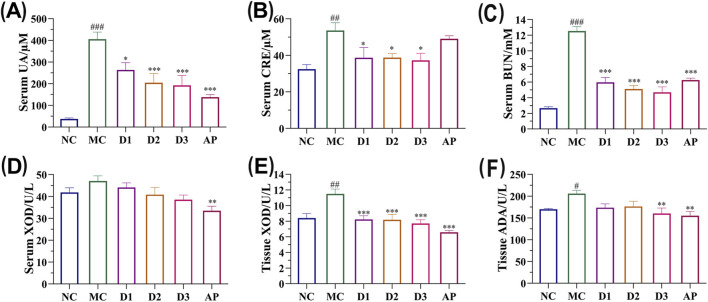
The effects of PLE-2 on the levels of UA. UA **(A)**, CRE **(B)**, BUN **(C)** and XOD **(D)** and the effect of PLE-2 on the levels of XOD **(E)** and ADA **(F)** in liver tissue homogenates of HUA rats. Data are from three independent experiments and expressed as mean ± SD (n = 6). Statistical analysis was performed using one-way ANOVA followed by Tukey’s *post hoc* test. Compared with the NC group, #p < 0.05, ##p < 0.01, ###p < 0.001; compared with the MC group, *p < 0.05, **p < 0.01, ***p < 0.001.

As shown in Figure 3A, continuous administration of PLE-2 significantly reduced UA levels in hyperuricemic rats, with a clear dose-dependent trend observed. With regard to renal protection, serum creatinine and BUN levels were significantly elevated in the model group (Figures 3B,C), confirming that HUA induced marked impairment of renal function. Following PLE-2 intervention, the reduction in serum creatinine was significantly greater than that achieved by the positive control drug AP (Figure 3B), indicating superior nephroprotective potential.

Figure 3D demonstrates a decreasing trend in XOD activity in the serum. Additionally, PLE-2 strongly inhibited XOD and ADA in liver tissues, with the high-dose group matching or exceeding AP’s efficacy (Figures 3E,F). PLE-2 demonstrates a dose-dependent reduction in serum UA levels, ameliorates renal impairment, and effectively inhibits key enzymes involved in UA synthesis.

### 3.4 Renal transporters and enzymes at mRNA and protein

**FIGURE 4:**
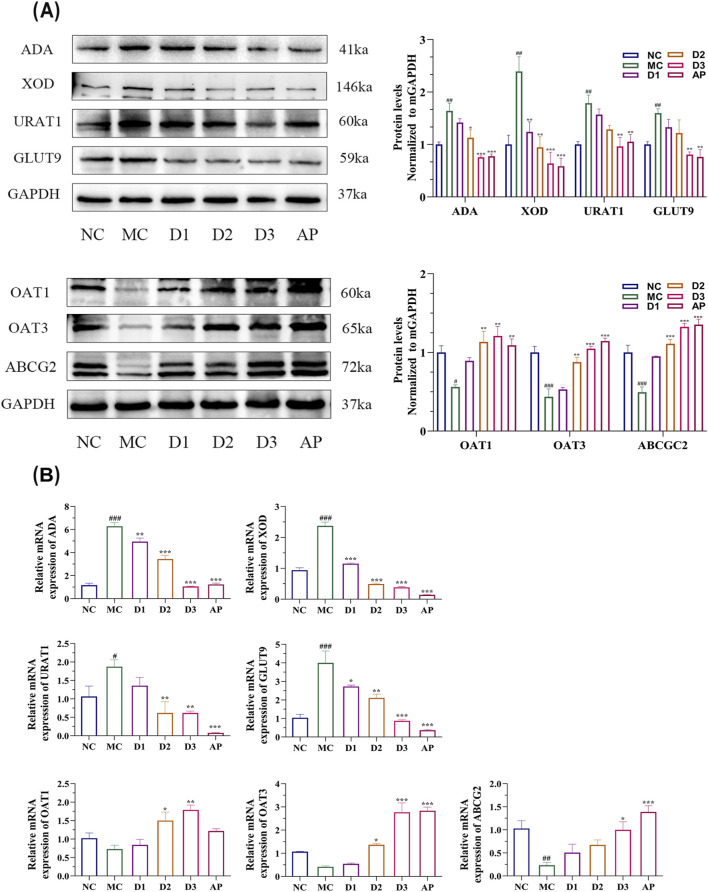
Renal tissue Western blot and qPCR analyses illustrate PLE-2-mediated regulation of UA transporters and metabolic enzymes in hyperuricemic rats. **(A)** Effects of PLE-2 and AP on the expression levels of enzymes and transport proteins involved in UA synthesis, including ADA, XOD, URAT1, GLUT9, OAT1, OAT3, and ABCG2 of HUA rats. **(B)** The qPCR results of metabolic enzymes and renal transport proteins. Data are from three independent experiments and expressed as mean ± SD (n = 6). Statistical analysis was performed using one-way ANOVA followed by Tukey’s *post hoc* test. Compared with the NC group, ##p < 0.01, ###p < 0.001; compared with the MC group, *p < 0.05, **p < 0.01, ***p < 0.001.

Figure 4 presents the mRNA and protein expression levels of XOD and ADA in kidney tissues. Both qPCR and Western blot analyses indicate that, compared with the normal control group, the mRNA and protein expression levels of XOD and ADA were significantly upregulated in the model group, consistent with the elevated serum UA levels observed in this group. Following pharmacological intervention, all treatment groups exhibited varying degrees of downregulation in the mRNA and protein expression of XOD and ADA, with the high-dose group showing the most pronounced reduction (P < 0.01). The PLE-2 coordinately suppresses the expression of UA synthesis-related enzymes at both transcriptional and translational levels, thereby underscoring the consistency and reliability of its mechanism of action.

In addition, abnormal expression of the renal UA transporters is also a key factor contributing to HUA. As shown in Figures 4A,B, compared with the model group, the tested drug treatment exhibited a bidirectional regulatory effect: on the one hand, it significantly downregulated the mRNA and protein levels of URAT1 and GLUT9, reducing UA reabsorption; on the other hand, it significantly upregulated the expression of OAT1, OAT3 and ABCG2, promoting UA excretion (Eckenstaler and Benndorf, 2021). The results of *in vivo* and *in vitro* experiments are consistent.

### 3.5 Histopathology assays

**FIGURE 5:**
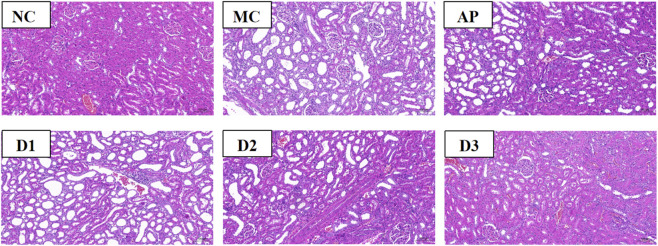
Histopathology assays of the kidney tissue. Representative H&E-stained sections illustrating the protective effects of PLE-2 against urate-induced renal injury across different experimental groups (NC, MC, AP, and PLE-2 treatments). Magnification: ×200; scale bar = 100 µm.

As shown in Figure 5, H&E staining revealed characteristic pathological changes in the model group, including degeneration and necrosis of renal tubular epithelial cells, tubular atrophy and dilation, as well as interstitial inflammatory infiltration, thereby confirming successful model establishment. The test drug showed a significant dose-dependent protective effect. While the low-dose group exhibited minimal improvement, the medium-dose group showed marked suppression of inflammation and amelioration of structural damage, with therapeutic efficacy comparable to that of the positive control group. The high-dose group displayed the most pronounced protective effect, characterized by only mild cellular edema and absence of significant structural disruption or inflammatory infiltration, indicating near-complete restoration of renal histological architecture (Nguyen et al., 2024). PLE-2 exerts its protective effects by suppressing inflammatory responses and maintaining renal tubular integrity.

### 3.6 Molecular docking results

HPLC fingerprint analysis indicated that paeoniflorin is the principal metabolite of *P. lactiflora*, a metabolite widely used as a quantitative marker for quality control in the Chinese Pharmacopoeia. Accordingly, paeoniflorin was selected as the starting point for virtual screening. As shown in the molecular docking results in Table 1 that the binding affinities between paeoniflorin and all tested targets were negative, suggesting potential binding interactions. Paeoniflorin exerts inhibitory effects by binding to UA-synthesizing enzymes (ADA and XOD) and simultaneously interacts specifically with transporters involved in UA reabsorption (URAT1 and GLUT9) and excretion (OAT1, OAT3, and ABCG2). These findings highlight its multi-target potential in modulating UA homeostasis at the molecular level, thereby providing a rational basis for further investigation into the bioactive metabolites of *P. lactiflora*. Furthermore, to rule out the possibility of artifactual readouts typically caused by pan-assay interference metabolites (PAINS) (Bolz et al., 2021; Magalhães et al., 2021), we computationally evaluated the liability of paeoniflorin. Analysis using the SwissADME web tool confirmed that the molecular scaffold of paeoniflorin triggers exactly 0 PAINS alerts and 0 Brenk alerts.

**TABLE 1:** | XXcategory

Target category	Target protein and UniProt ID	Binding affinity (kcal/mol)	Key interaction types and residues
UA synthase	ADA (ID: P00813)	-6.815	Strong H-bonds (LYS90, LEU304), Salt bridge (LYS23)
UA synthase	XOD (ID: P47989)	-6.522	Dense H-bond network, Salt bridge (LYS95)
Reabsorption transporter	URAT1 (ID: 9B1G)	-8.808	H-bonds, Salt bridge, Cation-π interaction (ARG473)
Reabsorption transporter	GLUT9 (ID: 8Y65)	-9.268	Strong hydrophobic driving (PHE451), Backbone H-bond (PHE435)
Excretion transporter	OAT1 (ID: 9B02)	-9.268	Hydrophobic pocket, Salt bridge (ARG466), Multiple H-bonds
Excretion transporter	OAT3 (ID: 6VXI)	-6.724	Dense polar H-bond network, Key salt bridge (LYS89)
Excretion transporter	ABCG2 (ID: 7V96)	-6.818	Aromatic hydrophobic pocket, Dual-function H-bond (PHE547)

## 4 Discussion

Our findings demonstrate that PLE-2 reduces serum UA levels by modulating two key pathways of UA homeostasis: hepatic synthesis and renal excretion. In HK-2 cells, PLE-2 mitigated UA-induced injury, suggesting it may interrupt the vicious cycle between elevated UA and renal damage. In the rat HUA model, oral PLE-2 (100, 200, and 400 mg/kg) lowered serum UA in a dose-dependent manner to levels comparable with allopurinol, while improving renal function (reduced creatinine and blood urea nitrogen) and kidney histology. These findings suggest that PLE-2 is a functional food metabolite with high UA-lowering efficacy and low adverse effects, offering a safe and effective natural alternative to synthetic pharmacotherapies (Chen et al., 2015; Mehmood et al., 2019).

Compared with single-target XOD inhibitors such as allopurinol, PLE-2 inhibits both XOD and ADA, two consecutive rate-limiting enzymes in the hepatic UA synthesis pathway. ADA converts adenosine to inosine (subsequently metabolized to hypoxanthine), while XOD oxidizes hypoxanthine and xanthine to UA. By targeting both enzymes, PLE-2 reduces the upstream supply of purine substrates and inhibits terminal UA production, leading to more comprehensive suppression of urate biosynthesis. Molecular docking predicted interactions between paeoniflorin (the major metabolite in PLE-2) and the catalytic sites of XOD and ADA, with hydrogen bonds formed at key residues. These observations are consistent with the experimental reductions in enzyme activity and provide a structural basis for PLE-2’s effects on endogenous UA synthesis. It should be noted that allopurinol, as a classical XOD inhibitor, acts primarily through direct inhibition of XOD enzymatic activity—not by directly suppressing XOD protein expression. The observed downregulation of XOD protein expression in the allopurinol-treated group in this study reflects an indirect, compensatory adaptation: hyperuricemic conditions induce metabolic stress and inflammatory responses, which can trigger compensatory upregulation of XOD expression. By markedly lowering serum UA levels, allopurinol alleviates HUA-associated pathological stress, thereby reversing this compensatory upregulation and ultimately reducing XOD protein expression.

PLE-2 also modulates renal urate transporters: it downregulates reabsorption transporters (URAT1 and GLUT9) and upregulates secretion transporters (OAT1, OAT3, and ABCG2), as shown by qPCR and Western blot analyses. This bidirectional adjustment restores the balance between reabsorption and excretion, which is disrupted in HUA due to URAT1/GLUT9 overactivation and OATs/ABCG2 impairment. The transporter changes align with the observed serum UA reduction and improved renal histology, supporting a coordinated mechanism involving both synthesis inhibition and excretion enhancement.

Beyond UA reduction, PLE-2 exhibited renal protective effects. It reversed UA–induced injury in HK-2 cells and attenuated histopathological damage *in vivo* (reduced inflammatory infiltration and preserved tubular structure in a dose-dependent manner). These findings suggest that PLE-2 may exert protective effects through anti-inflammatory mechanisms, complementing its urate-lowering activity. The purified nature of PLE-2, enriched in monoterpene glycosides such as paeoniflorin (the highest peak abundance in the HPLC fingerprint), likely contributes to these effects by improving metabolite stability and reducing impurities compared to crude extracts. While paeoniflorin docking provides mechanistic insight, its role is part of a multi-metabolite profile in PLE-2.

Moreover, this study establishes and validates a quantitative correlation between *in vitro* (HK-2 cell) and *in vivo* (rat) experimental outcomes. *In vitro*, the drug elicited significant biological effects within a concentration range of 1.25–20 μg/mL. Given the reported oral bioavailability of paeoniflorin—3%–5% in rats—the predicted steady-state plasma concentrations following oral administration fall squarely within this experimentally determined effective *in vitro* range (Xiao et al., 2024). This pharmacokinetically informed alignment of exposure levels across models strengthens the physiological relevance of the *in vitro* findings and supports the conclusion that the observed regulation of transporters and metabolic enzymes in HK-2 cells reflects bona fide systemic responses to paeoniflorin *in vivo*.

However, this study has several limitations. First, PLE-2 was evaluated predominantly *in vitro* (HK-2 cells) and in a rat model of HUA; thus, clinical trials in humans are essential to validate its translational efficacy and safety (Blanchard and Smoliga, 2015; Xiao et al., 2024). Second, as a multi-metabolite botanical fraction, PLE-2 contains numerous bioactive metabolites—whose individual contributions and potential synergistic or antagonistic interactions remain incompletely characterized (Algradi et al., 2021; Li et al., 2021). Third, while allopurinol was used as the positive control for XOD–mediated UA synthesis inhibition (Algradi et al., 2021), benzbromarone served solely as a reference uricosuric agent—not as an experimental comparator—in this study. This design reflects a deliberate focus on mechanistic dissection: specifically, elucidating PLE-2’s dual regulatory action—simultaneous suppression of hepatic UA production (by XOD and ADA inhibition) and restoration of renal urate excretion (by coordinated modulation of URAT1, GLUT9, OAT1/OAT3, and ABCG2). Consequently, direct head-to-head comparison of PLE-2’s uricosuric potency against benzbromarone was intentionally omitted to avoid diluting mechanistic resolution with comparative complexity. To address this, future work will integrate benzbromarone as a dedicated uricosuric comparator, enabling rigorous, multi-dimensional evaluation of PLE-2’s pharmacological profile (Algradi et al., 2021). Further priorities include comprehensive *in vivo* pharmacokinetic characterization of PLE-2, functional validation of its key metabolite metabolites, and ultimately, well-designed clinical trials to establish its long-term efficacy (Lin et al., 2024), safety, and therapeutic utility in hyperuricemic patients.

In conclusion, our study demonstrates that the purified PLE-2 fraction modulates UA homeostasis through a dual mechanism: suppression of hepatic urate synthesis via XOD and ADA inhibition, and promotion of renal excretion via urate transporter modulation. These urate-lowering effects, coupled with substantial renal protective properties, highlight the potential of PLE-2 as a promising phytotherapeutic candidate and functional food metabolite for the comprehensive management of HUA.

## 5 Conclusion

This study systematically characterized the phytochemical profile of the purified PLE-2 fraction isolated from the roots of *P*. *lactiflora* and rigorously demonstrated its potent hypouricemic and renoprotective effects in a well-established HUA model. The therapeutic efficacy of PLE-2 is driven by a dual-regulatory mechanism: the suppression of hepatic UA synthesis via XOD and ADA inhibition, coupled with the enhancement of renal urate excretion through the coordinated modulation of reabsorption (URAT1, GLUT9) and secretion (OAT1/3, ABCG2) transporters. Furthermore, PLE-2 effectively ameliorated HUA-induced renal histological damage and functional decline. Driven by the enrichment of active monoterpene glycosides, particularly paeoniflorin, these findings provide a solid pharmacological foundation for developing PLE-2 as a standardized, multi-target botanical candidate or functional food metabolite for the management of HUA and its associated renal complications.

## Data Availability

The raw data supporting the conclusions of this article will be made available by the authors, without undue reservation.
