# Supplementary material for: Dual mechanisms of a purified root extract of Paeonia lactiflora Pall. in regulating uric acid homeostasis: inhibition of hepatic synthesis and promotion of renal excretion in hyperuricemic rat models
Source: Front Pharmacol. 2026 Apr 7;17:1800063. doi: 10.3389/fphar.2026.1800063 (PMC13095781; doi:10.3389/fphar.2026.1800063)
Supplement: Supplementary file 1 [file Supplementaryfile1.pdf]

Supplementary Materials

Dual Mechanisms of a Purified Root Extract of *Paeonia lactiflora* Pall. in Regulating Uric Acid Homeostasis: Inhibition of Hepatic Synthesis and Promotion of Renal Excretion in Hyperuricemic Rat Models

Tingting Jin[†], Ruoling Xu[†], Zhuoxin Jiang, Juncheng Ma, Ning Li*

[†]These authors contributed equally to this work and share first authorship.

Anhui Key Laboratory of Bioactivity of Natural Products, School of Pharmacy, Anhui Medical University, Hefei 230032, China.

***Correspondence:**
Ning Li
E-mail: 1993500019@ahmu.edu.cn
Tel: +86-551-65161015

1 Supplementary Figures and Tables

1.1 Supplementary Table

S1. RT-qPCR primer sequences used in this study

Gene name	Sequences (5' to 3')
OAT3-F	CTGGCTACAGTTAACCTCGTCT
OAT3-R	CCTTCCTCCTTCTTGCCGTTG
ABCG2-F	AGAGCCTGTATATGTTACCTCGTT
ABCG2-R	AAGGGCACCAATAATCAGTCC
ADA-F	ATTCAACAAGCCCAAAGTAGAGC
ADA-R	CCGATAATGTTGCGTAGTCCCT
GLUT9-F	CTCGTGATTGAACCCGTAGCTC
GLUT9-R	CTGCCATTTCCAGTACAATGAGTG
OAT1-F	CAAGGACAACCCGAATCTTGC
OAT1-R	CATAGACCCAGCCATCAATGC
URAT1-F	ACAATAGCTGCCCTCATGCC
URAT1-R	GCATATAGCCTTCCCTACCCAG
XOD-F	GTTTGCTTCCTCACCGCAGA
XOD-R	TGCCCAACACAAGTAACCTCA
GAPDHF	GACATGCCGCCTGGAGAAAC
GAPDHR	AGCCCAGGATGCCCTTTAGT

1.2 Supplementary Figures

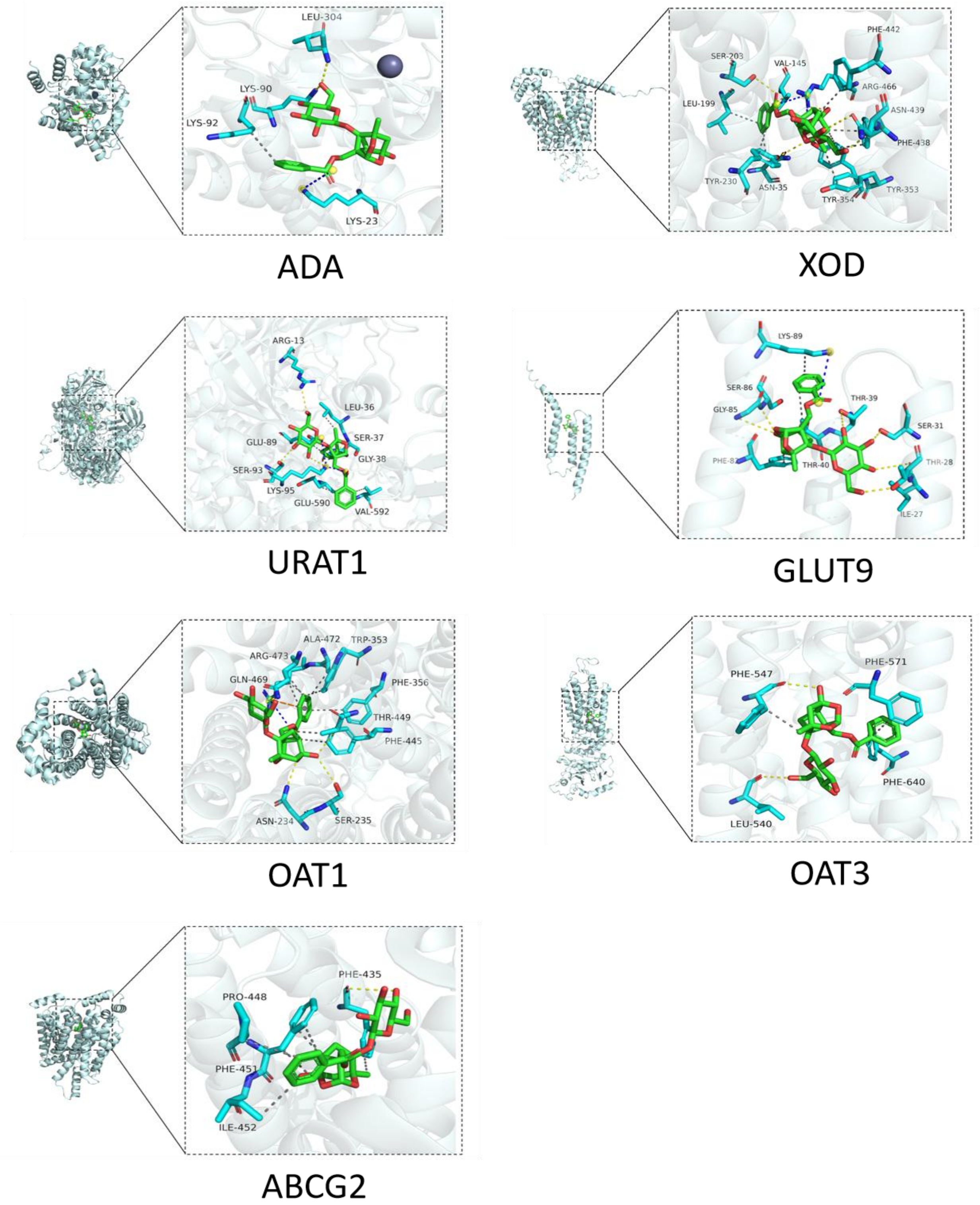

Figure S1. Molecular docking result diagram.In the diagram, the green sticks represent small molecules, the light cyan cartoon models depict proteins, yellow dashed lines indicate hydrogen bonding interactions, gray dashed lines denote hydrophobic interactions, blue dashed lines signify salt bridge interactions, and orange-yellow dashed lines represent π-cation stacking interactions.

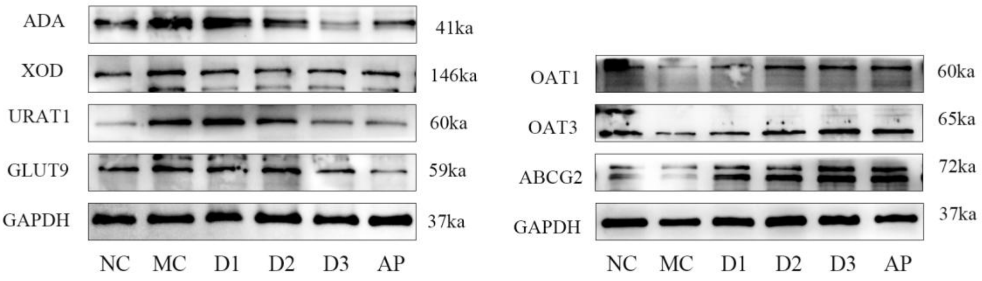

Figure S2. Cellular protein banding pattern diagram

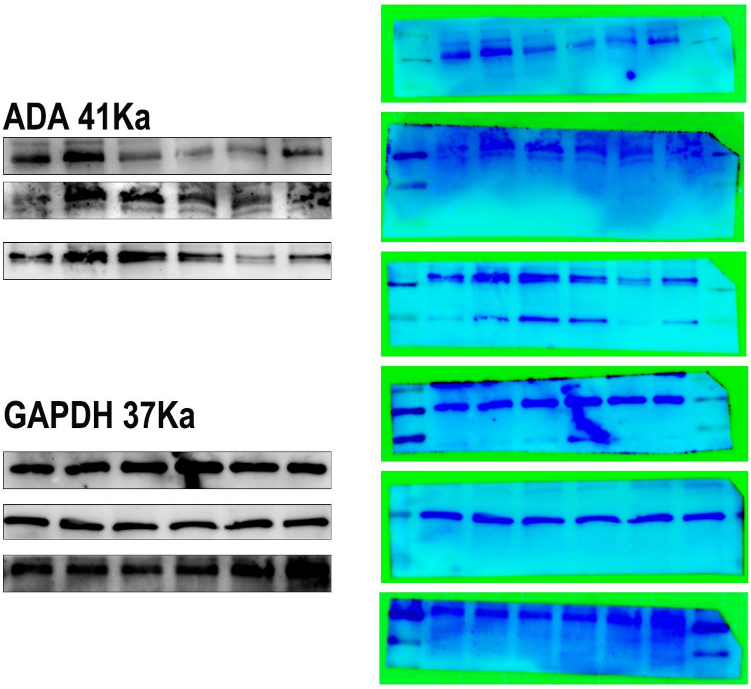

Figure S3. ADA protein banding pattern diagram

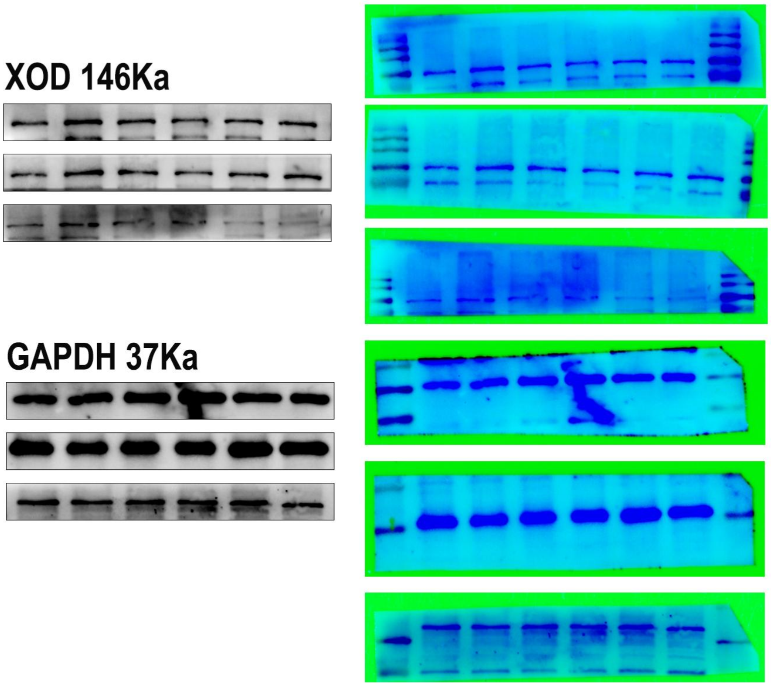

Figure S4. XOD protein banding pattern diagram

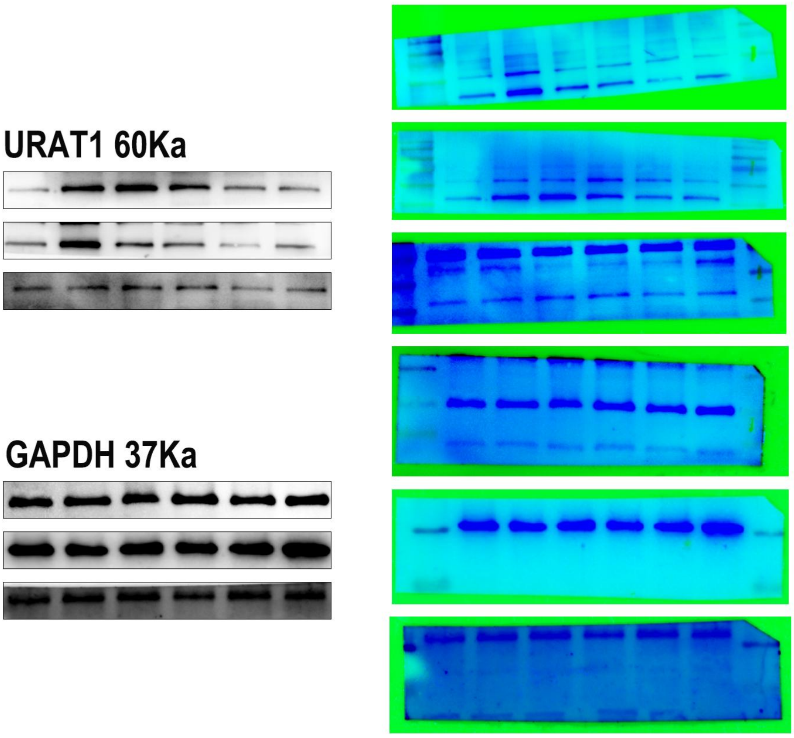

Figure S5. URAT1 protein banding pattern diagram

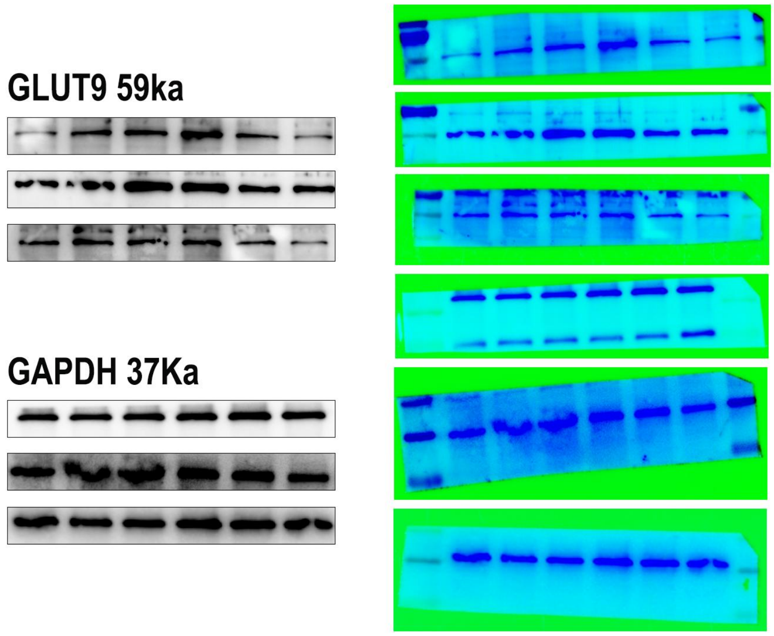

Figure S6. GLUT9 protein banding pattern diagram

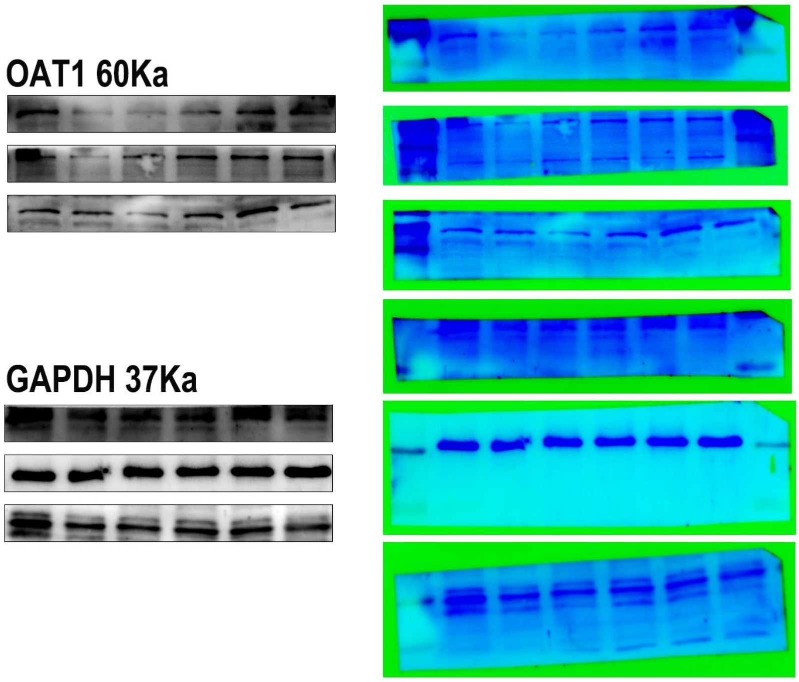

Figure S7. OAT1 protein banding pattern diagram

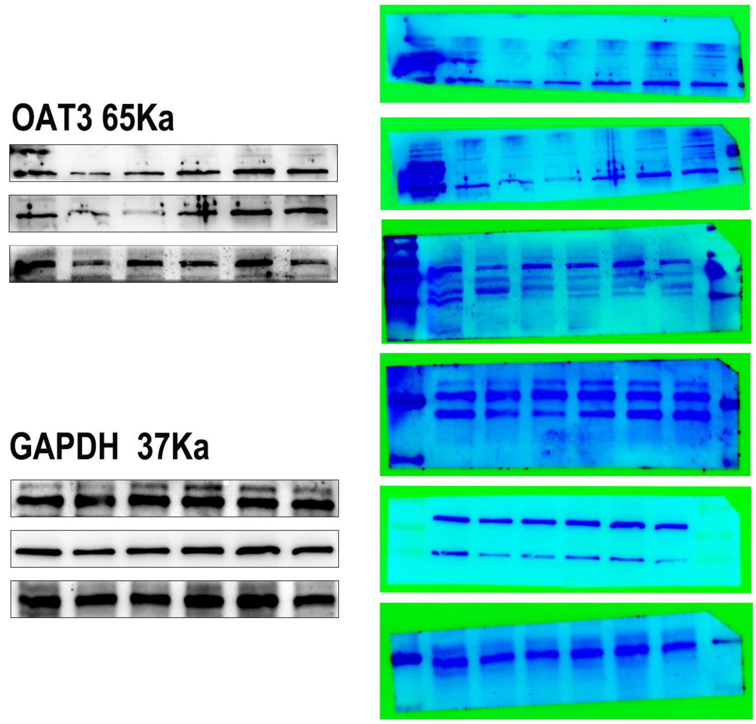

Figure S8. OAT3 protein banding pattern diagram

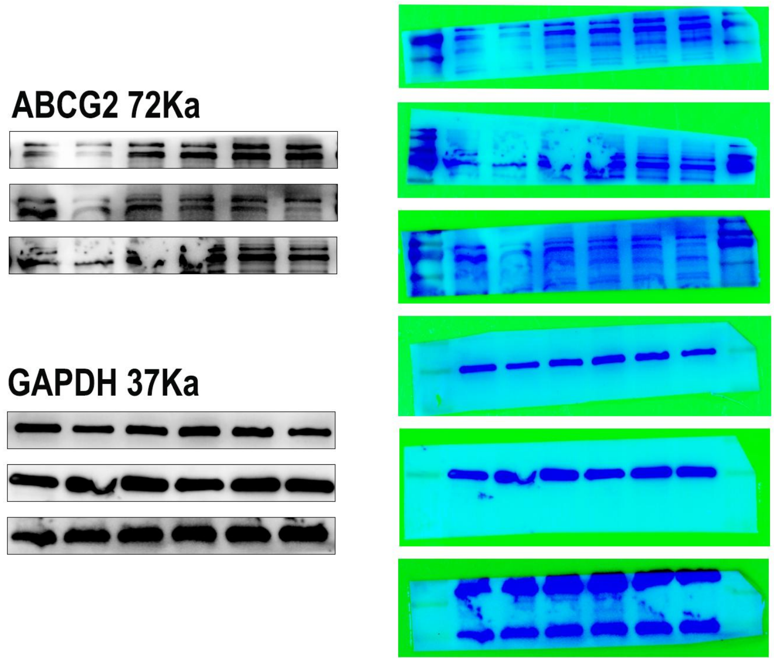

Figure S9. ABCG2 protein banding pattern diagram

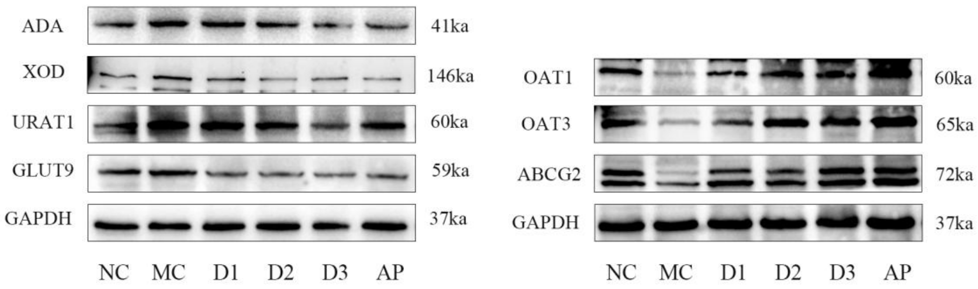

Figure S10. Organized protein band diagram.

Western blot analysis demonstrated that each experimental group contained n=6 biological replicates. The seventh lane was loaded with a non-experimental sample obtained from backup specimens, which was excluded from data analysis and graphical representation. For the uncropped blot images and complete lane annotations, please refer to Supplementary Figure S10.

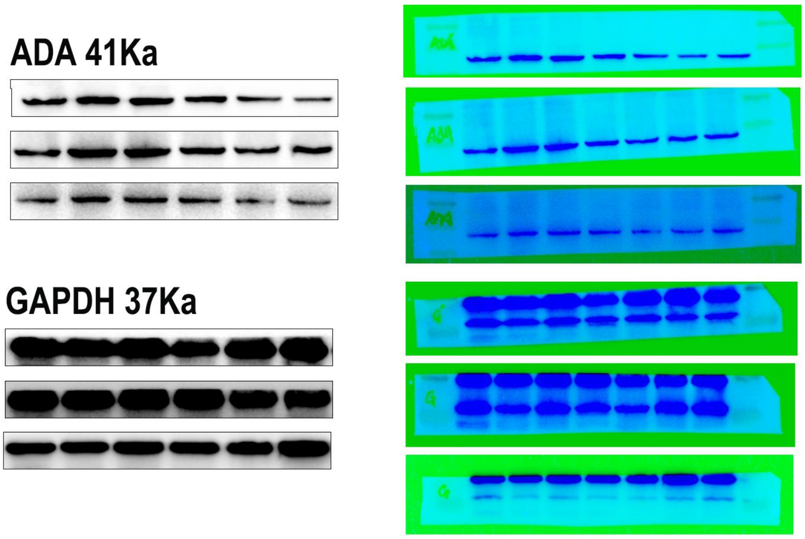

Figure S11. ADA protein banding pattern diagram

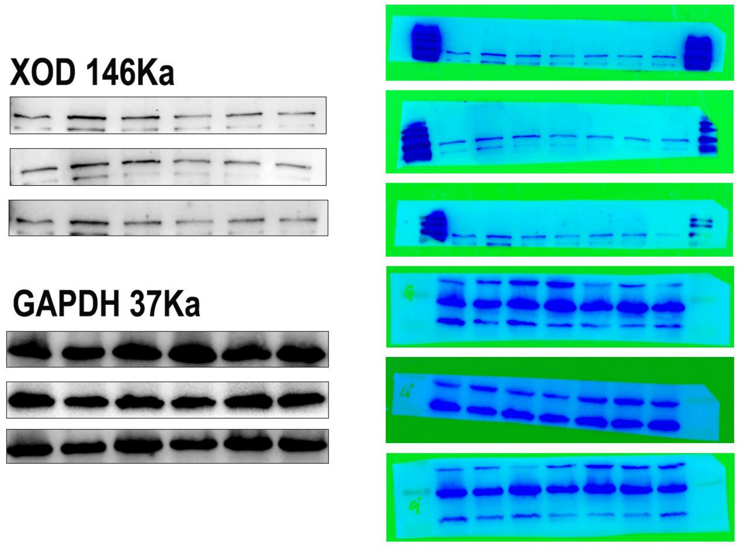

Figure S12. XOD protein banding pattern diagram

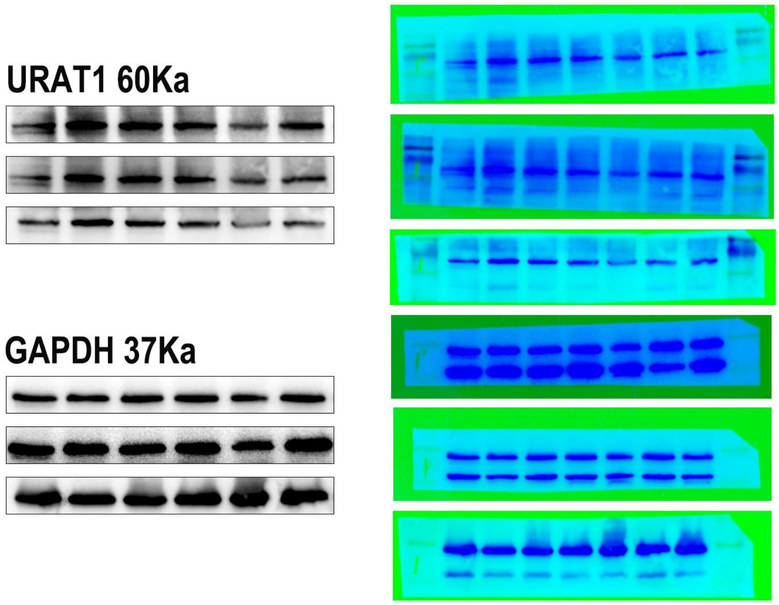

Figure S13. URAT1 protein banding pattern diagram

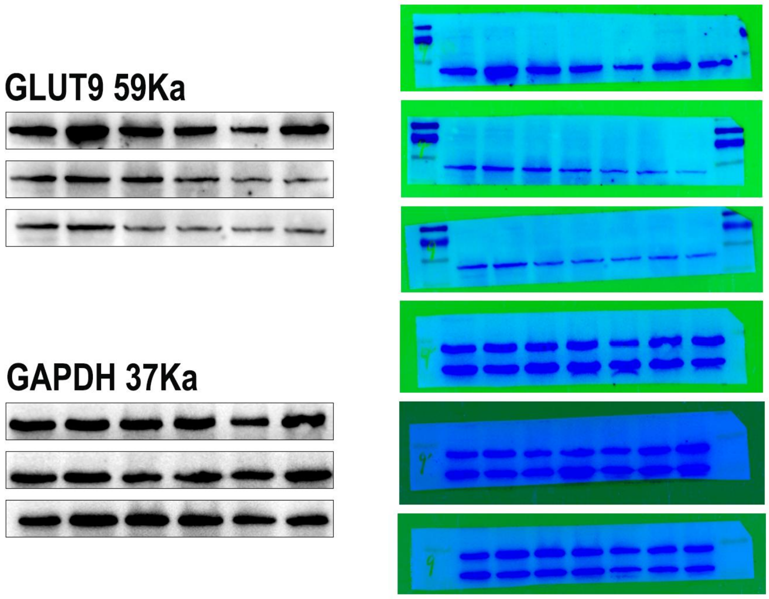

Figure S14. GLUT9 protein banding pattern diagram

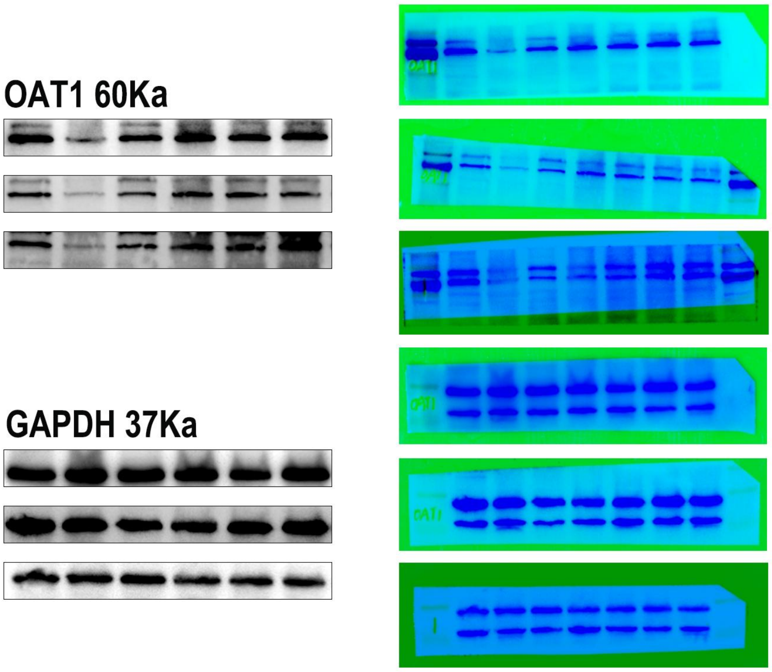

Figure S15. OAT1 protein banding pattern diagram

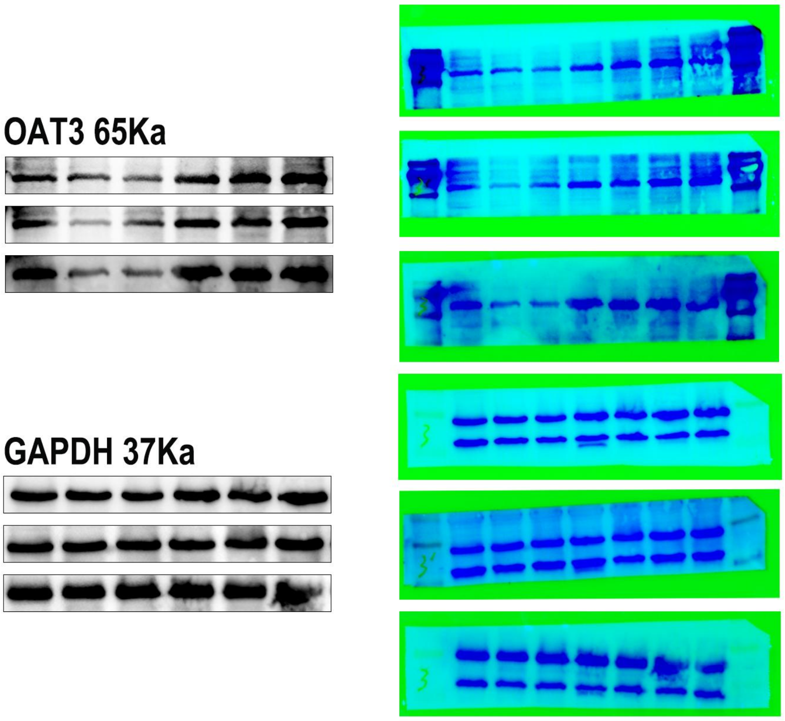

Figure S16. OAT3 protein banding pattern diagram

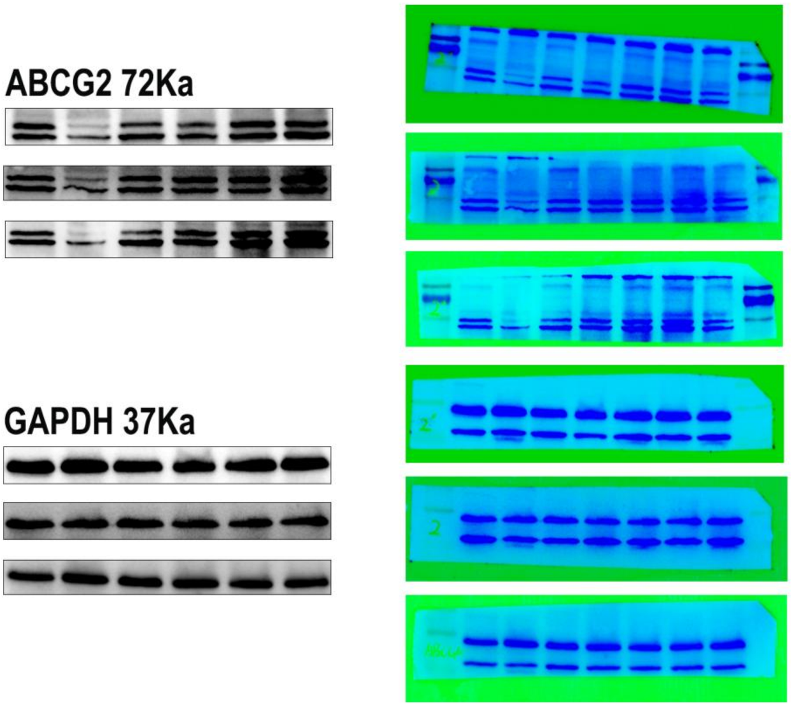

Figure S17. ABCG2 protein banding pattern diagram

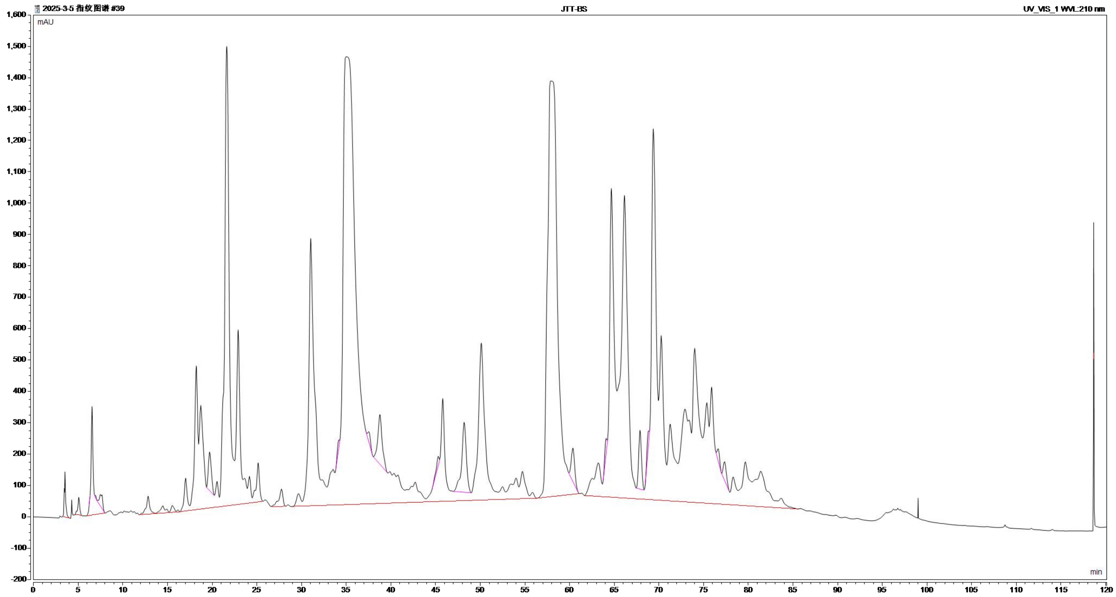

Figure S18. HPLC chromatograms of PLE-2 at 210nm detection wavelengths

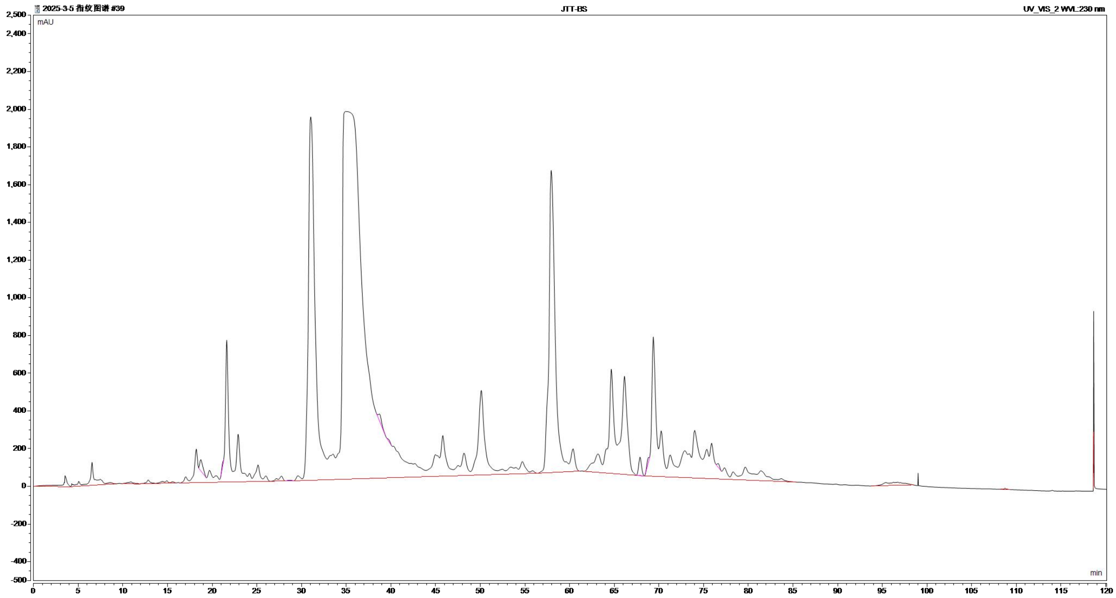

Figure S19. HPLC chromatograms of PLE-2 at 230nm detection wavelengths

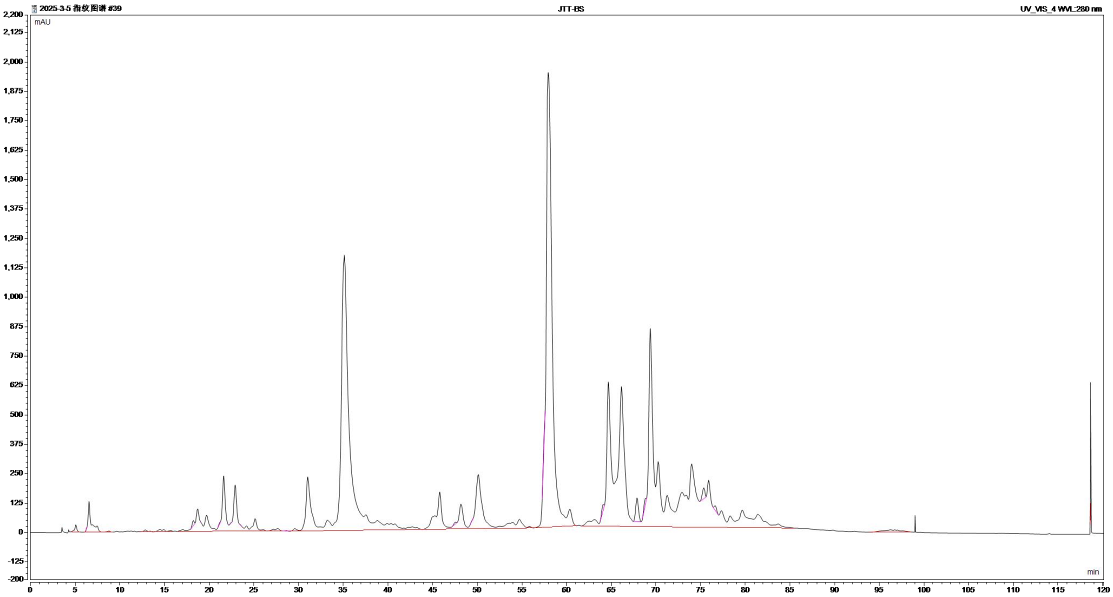

Figure S20. HPLC chromatograms of PLE-2 at 280nm detection wavelengths

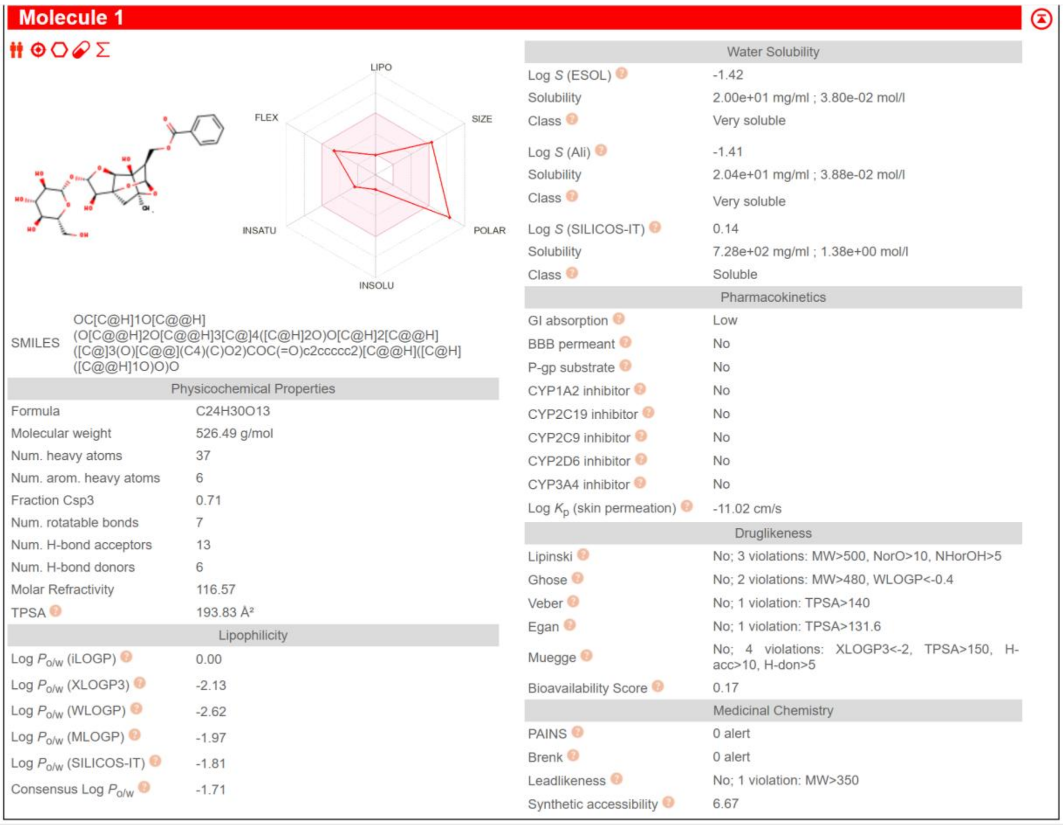

Molecule 1

SMILES: OC[C@H]1O[C@@H](O[C@@H]2O[C@@H]3[C@]4([C@H]2O)O[C@H]2[C@@H]([C@]3(O)[C@@](C4)(C)O2)COC(=O)c2ccccc2)[C@@H]([C@H]([C@@H]1O)O)O

Physicochemical Properties	
Formula	C24H30O13
Molecular weight	526.49 g/mol
Num. heavy atoms	37
Num. arom. heavy atoms	6
Fraction Csp3	0.71
Num. rotatable bonds	7
Num. H-bond acceptors	13
Num. H-bond donors	6
Molar Refractivity	116.57
TPSA	193.83 Å²

Lipophilicity	
Log $P_{o/w}$ (iLOGP)	0.00
Log $P_{o/w}$ (XLOGP3)	-2.13
Log $P_{o/w}$ (WLOGP)	-2.62
Log $P_{o/w}$ (MLOGP)	-1.97
Log $P_{o/w}$ (SILICOS-IT)	-1.81
Consensus Log $P_{o/w}$	-1.71

Water Solubility	
Log *S* (ESOL)	-1.42
Solubility	2.00e+01 mg/ml ; 3.80e-02 mol/l
Class	Very soluble
Log *S* (Ali)	-1.41
Solubility	2.04e+01 mg/ml ; 3.88e-02 mol/l
Class	Very soluble
Log *S* (SILICOS-IT)	0.14
Solubility	7.28e+02 mg/ml ; 1.38e+00 mol/l
Class	Soluble

Pharmacokinetics	
GI absorption	Low
BBB permeant	No
P-gp substrate	No
CYP1A2 inhibitor	No
CYP2C19 inhibitor	No
CYP2C9 inhibitor	No
CYP2D6 inhibitor	No
CYP3A4 inhibitor	No
Log K_p (skin permeation)	-11.02 cm/s

Druglikeness	
Lipinski	No; 3 violations: MW>500, NorO>10, NHorOH>5
Ghose	No; 2 violations: MW>480, WLOGP<-0.4
Veber	No; 1 violation: TPSA>140
Egan	No; 1 violation: TPSA>131.6
Muegge	No; 4 violations: XLOGP3<-2, TPSA>150, H-acc>10, H-don>5
Bioavailability Score	0.17

Medicinal Chemistry	
PAINS	0 alert
Brenk	0 alert
Leadlikeness	No; 1 violation: MW>350
Synthetic accessibility	6.67

Figure S21. SwissADME Analysis of Paeoniflorin Showing No PAINS or Brenk Alerts

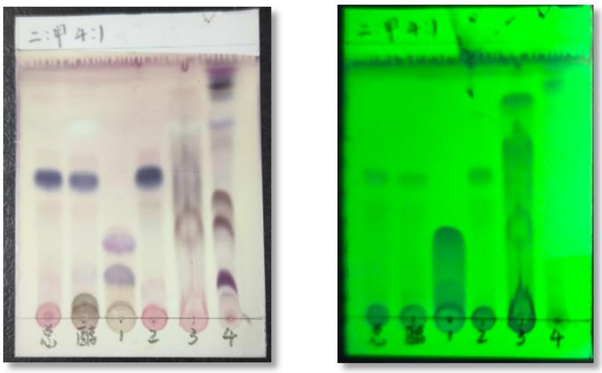

Figure S22. Thin-layer chromatography (TLC) comparison of total glucosides of paeony capsules, crude paeony extract, and different purified fractions from Paeonia lactiflora Pall.Mobile phase: dichloromethane–methanol (4:1, v/v). Samples: total glucosides of paeony capsules (“总”), crude paeony extract (“醇”), purified fractions PLE‑1 (“1”), PLE‑2 (“2”), PLE‑3 (“3”), and PLE‑4 (“4”). After visualization, spots were observed under visible light. PLE‑2 showed a deeper and more distinct spot corresponding to paeoniflorin, indicating higher purity and enrichment.

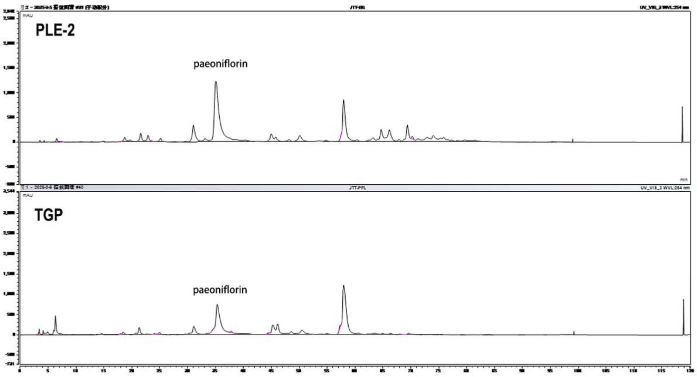

Figure S23. Comparison of HPLC fingerprints between PLE‑2 and total glucosides of paeony capsules (TGP).

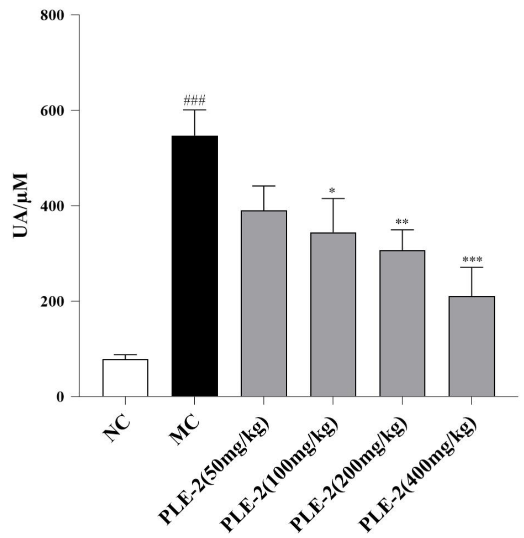

Figure S24. Dose-dependent effects of PLE-2 on serum uric acid levels in hyperuricemic SD rats. Rats were orally administered PLE-2 at doses of 50, 100, 200, and 400 mg/kg, respectively, and serum uric acid levels were measured to identify the optimal effective dose. Data are presented as mean ± standard deviation (n=6 per group).Compared with the NC group, ### $p < 0.001$; compared with the MC group, * $p < 0.05$, ** $p < 0.01$, *** $p < 0.001$.

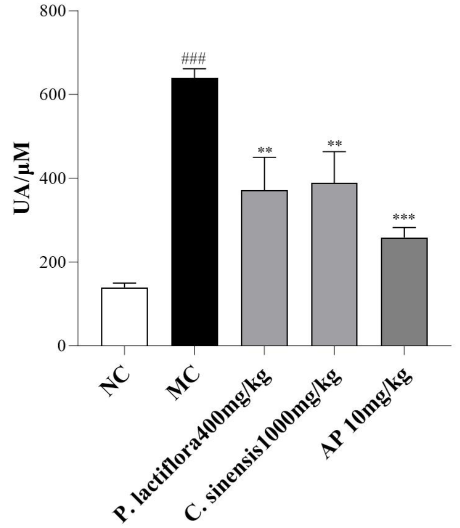

Figure S25. Screening effects of crude extracts from *P. lactiflora* and C. sinensis on serum uric acid in hyperuricemic rats.
